# Abnormal fingernail beds following carbon monoxide poisoning: a case report and review of the literature

**DOI:** 10.1186/1752-1947-8-263

**Published:** 2014-07-29

**Authors:** Mehrangiz Hatami, Frederick Naftolin, Masood A Khatamee

**Affiliations:** 1 Department of Obstetrics and Gynecology, School of Medicine, Shaheed Beheshti University of Medical Sciences and Health Services, Evin ST, Shahid Chamran Exp. Way, Tehran, Iran; 2 Department of Obstetrics and Gynecology, School of Medicine, New York University, 550 First Avenue, New York, NY 10016, USA

**Keywords:** Carbon monoxide poisoning, Carbon monoxide detector, Carboxyhemoglobin, Fingernail beds

## Abstract

**Introduction:**

Carbon monoxide poisoning is a very common cause of death in accidental, suicidal, or homicidal cases throughout the world. Fingernail bed manifestation is reported in survivors of carbon monoxide poisoning.

**Case presentation:**

A 40-year-old Caucasian woman was exposed to carbon monoxide when she was sleeping alone in her one-bedroom apartment; fortunately, the beeps from her First Alert combination smoke and carbon monoxide detector woke her and she was saved from any extensive health issues. The most indicative symptoms experienced were a severe headache, blurred vision, agitation, and confusion. Following contact with the Emergency Responses Services, she was promptly transferred to the hospital via ambulance and was treated with high-flow oxygen on the way. She was discharged from the emergency department on the same day, but carbon monoxide exposure had already had adverse effects on her fingernail beds. The fingernail tips were altered and appeared as if a bite had been taken out of their distal borders. The changes in the tips of her fingernails were significant, but they completely disappeared eight weeks later without any additional treatment.

**Conclusions:**

Worldwide, carbon monoxide poisoning is a potentially lethal condition that is preventable with educational programs and installation of carbon monoxide detectors in the home setting. Exposure to carbon monoxide frequently goes unrecognized until it is too late and causes silent death. To the best of the authors’ knowledge, this is the first report in the literature of fingernail bed manifestations in a survivor of carbon monoxide poisoning.

## Introduction

Carbon monoxide (CO) is a colorless, tasteless, odorless and nonirritating gas [1,2]. It has a high affinity for hemoglobin, 200 to 250 times more than hemoglobin's affinity for oxygen. Carbon monoxide binds to hemoglobin, forming carboxyhemoglobin, and can shift the oxygen-hemoglobin dissociation curve to the left [2-4].

Carboxyhemoglobin decreases the oxygen delivery to essential organs and tissues, and causes hypoxia, which results in death. The brain, heart, lungs, kidneys, and other organs that are more susceptible to hypoxia may be critically damaged by carbon monoxide exposure [5]. Signs and symptoms of carbon monoxide poisoning are vague and may be mistaken for a migraine, cerebrovascular accident, psychiatric illness, depression, Parkinson’s disease, gastroenteritis, chronic fatigue syndrome, influenza, alcohol intoxication, and heart disease [2,3]. We describe the first case of acute carbon monoxide poisoning with the fingernail bed effect mentioned above and review the literature regarding carbon monoxide poisoning.

## Case presentation

A 40-year-old Caucasian woman, a physician, was exposed to carbon monoxide in her one-bedroom apartment. She woke up at 3 a.m. with a severe headache, disturbed by the unusual intermittent beeps that were produced by the First Alert combination smoke and carbon monoxide detector that she had installed three months previously. She called 911 and promptly opened all of the six windows of her apartment, based on the operator’s suggestion. When New York Fire Department (NYFD) team reached her apartment, the gas supply was controlled and was turned off but the beeps from detector, accompanied with flashes and the blinking of red lights, did not cease until the batteries of the device were taken out. The NYFD found carbon monoxide (CO) levels 60 parts per million (ppm) in her home. The presentation CO level was 3.9 percent. At that moment, her blood pressure was 150/100mmHg, heart rate was 88 beats per minute, respiratory rate was 18 breaths per minute, and her temperature was 97.6°F. She had a severe headache in the frontal area, felt deep pain in the orbits, and had blurred vision, light-headedness and stabbing chest pain. She was agitated and her concentration was decreased.

**Figure 1:**
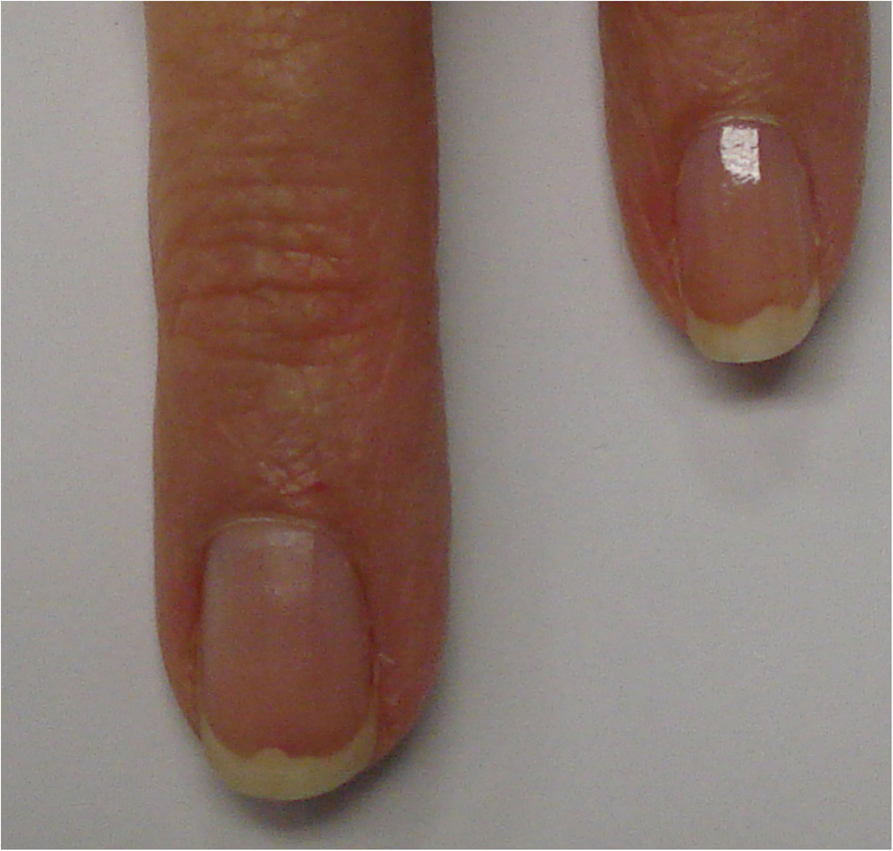
Effects of carbon monoxide poisoning on the fingernails on the first day of exposure.

**Figure 2:**
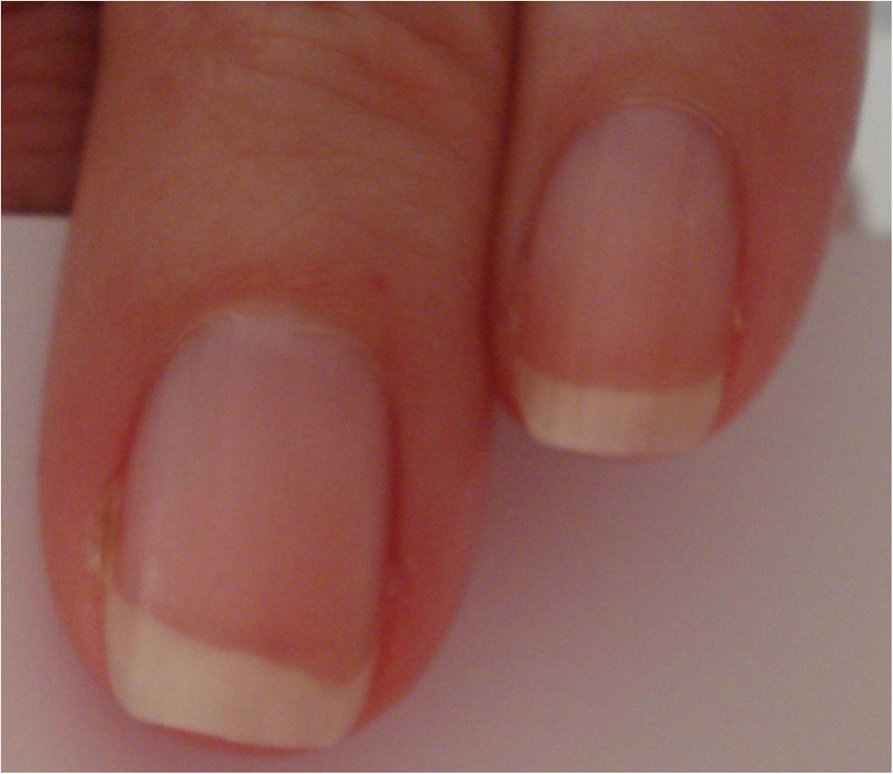
Fingernail beds appearance one month after carbon monoxide exposure.

**Figure 3:**
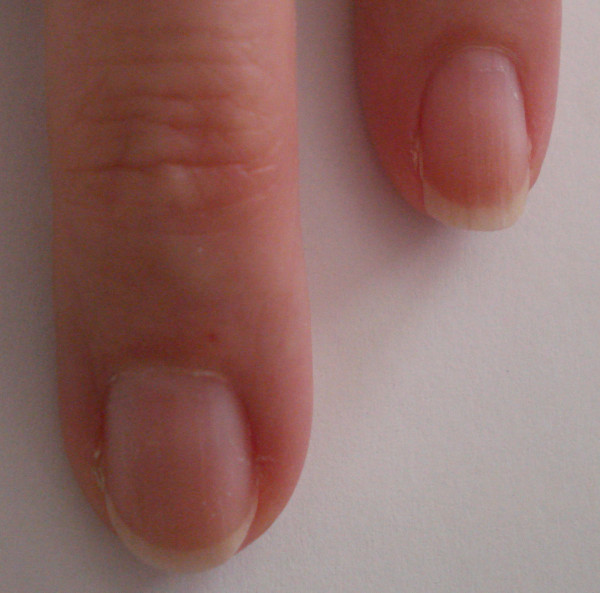
Fingernail beds appearance two months after carbon monoxide intoxication.

The patient has no previous history of headaches, except for a similar experience three months previously when she woke up at 3 a.m. with the same symptoms, which were related to the severe continuous flow of gas from the radiator placed toward her bed. When she opened the windows, the flow of gas stopped. She was able to breathe fresh air. On the same day, when she was looking for covers for the four radiators in her apartment, the salesperson at the hardware store recommended that she buy a carbon monoxide detector. Except for this incident, she was in good health condition without any past medical illness.The patient was immediately transferred to the emergency department. While traveling, the patient was placed on a high flow of 100 percent oxygen via a mask at normal atmospheric pressure. Carbon monoxide exposure was the diagnosis. After treatment with oxygen, her vital signs were within normal limits and her blood pressure decreased from 150/100 to 140/70mmHg. She opened her eyes completely, made spontaneous movements of all extremities, became more alert, and was able to respond coherently to the questions. She was discharged from the emergency department on the same day and her headache improved the day after, but some areas at the top margin of her fingernails were completely destroyed and marked with a bite out of the border without any separation between nail bed and nail plate. The nail bed border under her fingernails showed areas of erosion instead of a curved pattern as shown in Figure 1. These changes occurred in all fingernail beds but prominently in the fingernails of the first and second fingers. She denied any history of trauma to her fingernails or extremities. There were no other problems such as hemorrhage, white lines, spots or deformities on the surface of the nails. The distal edge, proximal end, and lateral margins of the nails were completely normal without any evidence of paronychial infection. The fingernail beds’ color was pink, and redder and bluish at the top border. There was no pain in the nails. At the time of the 10-month follow-up examination, the nails had returned to normal (Figure 2 and Figure 3).

## Discussion

Carbon monoxide poisoning is one of the most serious health problems worldwide and refers to the exposure of carbon monoxide levels higher than 3 percent in nonsmokers [2,3]. Fetuses, infants, older persons (especially those with cardiopulmonary diseases), and pregnant women (because of fetal hemoglobin) have a higher affinity than adults for carbon monoxide; they are at more of a risk and show symptoms of toxicity at a lower level of carbon monoxide [2,3,5]. Prolonged exposure to a high level of carbon monoxide during gestation may produce abortion, still birth, low birth weight, delayed brain development, motor disability, microcephaly, and limb malformation [5]. In the United States, about 50,000 patients are exposed to carbon monoxide and transferred to the emergency department each year; there are 2700 to 3800 deaths and 1400 of them are accidental [2,6]. Carbon monoxide is produced by the incomplete burning of carbon-containing fuel (such as gas, charcoal, coke, oil, wood), ovens, fireplaces, boilers, gas-powered water heaters, car exhausts, charcoal grills paraffin heaters, propane gas cylinders, and tobacco smoke [4,6]. Methylene chloride (dichloromethane), with its narcotic and hypoxic effect, is another source of carbon monoxide. It is in the vapors of some paint strippers, sprays, and it is widely used as an industrial solvent. Dichloromethane is absorbed via the skin, lungs, and transformed into carbon monoxide in the liver, then accumulates in the body and is later released slowly, extending the carbon monoxide half-life by more than double [4,7].

As Ruth-Sahd *et al*. have mentioned, carbon monoxide could have an effect on cellular toxicity and by binding to other proteins such as myoglobin, cytochrome cyclase, and guanylyl cyclase, could interfere with cell metabolism. Inhibition of cytochrome cyclase results in systemic hypoxia, oxidative stress, cellular necrosis, apoptosis, and formation of oxygen free radicals. Inhibition of guanylyl cyclase causes cerebral vasodilation that is associated with loss of consciousness, syncope, and hypotension. Carbon monoxide also causes low perfusion and hypoxia by tissue damage that result in the migration of leukocytes and an inflammatory reaction that increases capillary leakage and edema. Hypoxia also causes myocardial damage, tachycardia, arrhythmias and myocardial ischemia. Ruth-Sahd *et al*. believe that tissue reoxygenation is associated with reperfusion injury and lipid peroxidation, which leads to reversible demyelination of the central nervous system lipids. Nitric oxide levels also increase on carbon monoxide exposure, which causes peripheral vasodilatation that is responsible for decreasing cerebral blood flow, resulting in systemic hypotension. Nitric oxide is largely converted to methemoglobin in the body, and accelerates free radical formation causing endothelial and oxidative damage [2]. Carbon monoxide poisoning is usually diagnosed by measuring carboxyhemoglobin in the blood, carbon monoxide in expired breath, or by the presence of clinical signs and symptoms [3,5]. Because the majority of oximeter specificities is not high and reads carboxyhemoglobin as oxihemoglobin, the pulse oximetry has a lower value in the diagnosis of this condition [8,9].

Manifestations of carbon monoxide poisoning symptoms depend on the duration of exposure and blood gas concentration. A headache could be found after two to three hours, one to two hours, or 45 minutes of exposure with the concentration of 200, 400, or 800ppm respectively; an increase in the concentration to 6400ppm for example, results in a more severe occurrence and a faster onset of symptoms. The patients could therefore get a headache after one to two minutes and die within 10 to 15 minutes. Early clinical findings may present as increased blood pressure, heart rate, and respiratory rate [2,5]. Henz and Maeder [4] have reported in a prospective study of accidental carbon monoxide poisoning in 38 Swiss soldiers that dizziness (92 percent) and headache (87 percent) had the highest incidence of symptoms within the first two weeks, followed by weakness (76 percent), nausea (71 percent), shortness of breath (36 percent), chest pain (34 percent), visual changes (34 percent), confusion (29 percent), palpitations (24 percent), auditory symptoms (24 percent), clumsiness (24 percent), vomiting (18 percent), and loss of consciousness (16 percent). The mild signs and symptoms of carbon monoxide poisoning are presented at the level of less than 30 percent carboxyhemoglobin and include headache, nausea, vomiting, drowsiness, dizziness and confusion, fatigue, palpitation, malaise, visual disturbances, rapid heartbeat, and muscle jerks [2,5,10,11] whereas the moderate signs and symptoms of carbon monoxide poisoning occur at the level of more than 30 percent to 40 percent carboxyhemoglobin and are associated with chest pain, dyspnea, tachycardia, and tachypnea. Severe symptoms, such as difficulty breathing on exertion, palpitations, hypotension, confusion, convulsions, paralysis, coma, and death are usually manifested at a level of greater than 40 percent carboxyhemoglobin [2,5].

Other clinical manifestations of carbon monoxide poisoning that have been reported by Choi [5] include gastrointestinal symptoms (peptic ulcer, bleeding, and hepatomegaly), endocrinology disorders (hyperglycemia, acute hyperthyroidism, adrenal effects), skin and skeletal muscle disorders (erythema, ulcer, gangrene, alopecia, osteomyelitis), visual disturbances (retinal hemorrhage, papilledema, retinopathy, optic atrophy, amblyopia, scotoma, hemianopsia, and blindness), hearing impairment, and hematological disorder (leukocytosis, erythrocytosis, anemia, thrombocytopenia). Carbon monoxide poisoning can also cause a decrease in the weight of the testes and the number of spermatozoa in rats and cause incontinence, dysmenorrhea, menorragia, and decreased libido in women.

The occurrence of carbon monoxide poisoning is higher in colder weather conditions when there is increased usage of gas and heating [2,3]. Although our patient did not have severe signs and symptoms of carbon monoxide poisoning and was discharged from the hospital without any subsequent permanent neurologic problems, it is reported that death during hospitalization is 26 percent [12]. The rate is decreased to 2.6 percent in patients medically treated with hyperbaric oxygen [6]. There are permanent neurologic problems in 46 percent of survivors and only 28 percent of patients completely recover [12]. Long-term damage to the brain and heart have been shown even after a single moderate to severe carbon monoxide exposure, abnormal magnetic resonance imaging (MRI), and hyperbaric oxygen therapy. However, delayed neurological manifestations such as cognitive and personality changes, incontinence, psychosis, and parkinsonism [13,14] develop between two days and eight months later in 10 percent to 30 percent of survivors that are improved in 50 to 75 percent of patients during a year [3,15]. Brain MRI scans usually show lesions affecting basal ganglia, globus pallidus and white matter changes within the corpus callosum and periventricular region [3,5,13]. Cardiac damage manifestation due to carbon monoxide poisoning is included in symptoms such as decreased myocardial function, vasodilatation, hypoxia, hypotension, tachycardia, chest pain, arrhythmia, supraventricular tachycardia [16], sinus tachycardia, atrial fibrillation, premature atrial complexes, conduction abnormalities, premature ventricular complexes, ventricular fibrillation, myocardial ischemia, infarction, and cardiac arrest [3,5]. Carbon monoxide poisoning can also cause respiratory alkalosis and metabolic acidosis, pulmonary edema, aspiration, rhabdomyolysis, kidney damage and renal failure, and peripheral cyanosis [3,5,6]. Carboxyhemoglobin is rarely associated with blood that is a cherry-red color and with abnormal red colored skin, lips, nail beds and mucous membranes [1,3].

Pathologic finding in the appearance and color of the fingernails may suggest an underlying disease. Disorders such as clubbing, koilonychias or ‘spoon-shaped’ nails often suggest bronchogenic carcinoma, inflammatory bowel disease, or anemia, respectively. Onycholysis may be associated with trauma, psoriasis, or hyperthyroidism, warts, and onychomycosis. The finding of Beau’s lines refers to severe systemic disease that disrupts nail growth, Raynaud’s disease, pemphigus, or trauma. Mee’s lines are found in arsenic poisoning, Hodgkin’s disease, congestive heart failure, leprosy, malaria, chemotherapy, and carbon monoxide poisoning. Muehrcke’s lines are specific for decreasing albumin levels less than 2g per dL and vanish when the protein levels return to normal. They also may be present in patients with nephrotic syndrome, liver disease, and malnutrition. Splinter hemorrhage could be associated with subacute bacterial endocarditis, systemic lupus erythematosus (SLE), rheumatoid arthritis, antiphospholipid syndrome, peptic ulcer disease, malignancies, oral contraceptive use, pregnancy, psoriasis, and trauma. Telangiectasia may be found in rheumatoid arthritis, SLE, dermatomyositis, and scleroderma. Koilonychia, or pitting of the nails, also may correlate with connective tissue disorders [17].

Carbone monoxide, arsenic, and silver poisoning may cause fingernail appearance or color abnormalities [1,17]. Therefore, during physical examination, nail findings may lead to the diagnosis of the disease, the differential diagnosis, and whether or not further workup is required. In the present case, no immediate change in the nail beds was reported. However, a transient loss of distal nail bed integrity evolved. This might be related to the extreme capillary vascularity in the nail beds. It is possible that binding carbon monoxide to hemoglobin, and therefore shifting the oxygen, caused changes in the fingernail bed circulation that affected the integrity of the distal tip of the fingernail beds. In spite of the color change, the top of the skin beneath the nail plate changed as well and showed a bitten area. It is supposed that in this situation after carbon monoxide poisoning, capillaries of the fingernail beds constricted and as a result, the tip of the fingernail beds that are usually semicircular are damaged as shown in Figures 1 and 2. They slanted downward, which might be due to local ischemia, hypoxia, and poor refill in some areas and may be followed by subsequent apoptosis. It seems the microvasculature of the dorsal fingertips as a result of hypoxia injury is responsible for these changes [18]. In contrary to the conditions that are known as ‘onycholysis’, in this situation the nail plate was not separated from the nail bed and there was not any abnormal space between nail bed and nail plate [17]. Carbon monoxide poisoning and carboxyhemoglobin causes pink and bright-red coloration of the skin, and mucosal membranes with a cherry-red appearance, that can be treated with oxygen [1,3]. Langlois reported that the fingernails of carbon monoxide victims tend to exhibit a pink color; the fingernails of a dead body change to a darker and bluish-red color due to the cells’ anaerobic metabolism, resaturation of hemoglobin, and shift in the oxygen-binding affinity in the low temperatures [1]. Awareness of carbon monoxide poisoning and detectors are very valuable and, as in the presented case, could prevent subsequent death [3,19,20]. Despite the fact that the installation of carbon monoxide detectors is a law in New York State, many of the buildings and apartments, especially in private houses, do not have them. Furthermore, sometimes residents that are new to the United States do not have enough knowledge and information about this problem. The results of a survey done by Hampson and Weaver [20] have confirmed that only 51 percent of Utah and Washington residents used carbon monoxide alarms. This device is not expensive and, if it is installed in the ceilings of bedroom areas, it can save lives, especially when someone lives alone. However, just by putting the device on the desk close to the bed, it sounded before the concentration of carbon monoxide reached critical levels and caused severe damage. As Abelsohn *et al*. have mentioned, carbon monoxide poisoning alarms will start sounding within three hours nine minutes when a 70ppm concentration of carbon monoxide is present in the environment, within 50 minutes when 150ppm, or within 15 minutes if 400ppm is present [3]. Upon hearing the beep of the carbon monoxide alarm, all of the windows should be opened and everyone should leave the area promptly; the fire department should be contacted immediately. In addition to patient education, the source of carbon monoxide must be identified before the patient is discharged and sent back home. Oxygen is the antidote of carbon monoxide. High-flow 100 percent oxygen via a tight-fitting, non-rebreather mask, or endotracheal tube is administered for treating mild carbon monoxide poisoning symptoms and approximately reduces the carboxyhemoglobin in 80 minutes [2,6,12,21]. This treatment is usually continued for six hours, or until carboxyhemoglobin levels are reduced to less than 5 percent [2,3] whereas hyperbaric oxygen, which is occasionally used for severe symptoms, reduces the carboxyhemoglobin in about 22 minutes [6]. Wright believes that if a patient is given oxygen during their transportation to the emergency department, it will be difficult to know when the carboxyhemoglobin level peaked [21].

## Conclusions

In conclusion, clinical manifestations such as abnormal fingernail beds should be considered in the diagnosis of carbon monoxide intoxication. In addition, for prevention of death or long-term sequelae following carbon monoxide poisoning, installation of a carbon monoxide detector, implementation of educational programs, and evaluation and resolution of the source of production of carbon monoxide are all strongly recommended.

## Consent

Written informed consent was obtained from the patient for publication of this manuscript and any accompanying images. A copy of the written consent is available for review by the Editor-in-Chief of this journal.

## Abbreviations

CO: carbon monoxide; NYFD: New York Fire Department; ppm: parts per million; MRI: magnetic resonance imaging; SLE: systemic lupus erythematosus.

## Competing interests

The authors declare that they have no competing interests.

## Authors’ contributions

All authors contributed, read and approved the final manuscript.
